# Enhancing membrane repair increases regeneration in a sciatic injury model

**DOI:** 10.1371/journal.pone.0231194

**Published:** 2020-04-09

**Authors:** Brian J. Paleo, Kathryn M. Madalena, Rohan Mital, Kevin E. McElhanon, Thomas A. Kwiatkowski, Aubrey L. Rose, Jessica K. Lerch, Noah Weisleder

**Affiliations:** 1 Department of Physiology and Cell Biology, Davis Heart and Lung Research Institute, The Ohio State University, Columbus, Ohio, United States of America; 2 Department of Neuroscience, The Ohio State University, Columbus, Ohio, United States of America; University of Houston, UNITED STATES

## Abstract

Various injuries to the neural tissues can cause irreversible damage to multiple functions of the nervous system ranging from motor control to cognitive function. The limited treatment options available for patients have led to extensive interest in studying the mechanisms of neuronal regeneration and recovery from injury. Since many neurons are terminally differentiated, by increasing cell survival following injury it may be possible to minimize the impact of these injuries and provide translational potential for treatment of neuronal diseases. While several cell types are known to survive injury through plasma membrane repair mechanisms, there has been little investigation of membrane repair in neurons and even fewer efforts to target membrane repair as a therapy in neurons. Studies from our laboratory group and others demonstrated that mitsugumin 53 (MG53), a muscle-enriched tripartite motif (TRIM) family protein also known as TRIM72, is an essential component of the cell membrane repair machinery in skeletal muscle. Interestingly, recombinant human MG53 (rhMG53) can be applied exogenously to increase membrane repair capacity both *in vitro* and *in vivo*. Increasing the membrane repair capacity of neurons could potentially minimize the death of these cells and affect the progression of various neuronal diseases. In this study we assess the therapeutic potential of rhMG53 to increase membrane repair in cultured neurons and in an *in vivo* mouse model of neurotrauma. We found that a robust repair response exists in various neuronal cells and that rhMG53 can increase neuronal membrane repair both *in vitro* and *in vivo*. These findings provide direct evidence of conserved membrane repair responses in neurons and that these repair mechanisms can be targeted as a potential therapeutic approach for neuronal injury.

## Introduction

A wide variety of neurodegenerative diseases and traumatic injuries can cause irreversible damage to neural tissue and compromise various functions of the nervous system. Due to limited treatment options after neurodegeneration, there are intensive efforts underway investigating the mechanisms of neuronal regeneration and recovery from injury. Despite such efforts, these processes are not yet fully understood, and this lack of knowledge has hampered the development of therapeutics in this area. Since many neurons are terminally differentiated and axonal regeneration can result in reinnervation of off-target tissue [1–3], emphasis on maintaining cell survival could provide increased translational potential for the treatment of neuronal injury. While several cell types are known to survive injury through plasma membrane repair mechanisms, there has been little investigation of membrane repair in neurons and even fewer efforts to target membrane repair in diseases affecting neurons [4–8]

Plasma membrane repair is a conserved mechanism observed in many cells from simple single cell eggs to most adult mammalian cell types [9]. Membrane repair mechanisms function to close disruptions in the plasma membrane to restore structural integrity and to maintain barrier function to prevent cell death following injury. These repair mechanisms allow cells to survive an injury, which has great advantages over regeneration of a new cell to replace a large, complex cell like a neuron. While disruptions in the plasma membrane measuring approximately 1 nm or less will reseal through thermodynamic rearrangement of the component phospholipids, larger injuries generally require an active repair response to restore membrane integrity [10].

The mechanical tension on the plasma membrane produced by the connections of the membrane to the cytoskeleton and extracellular matrix can force disruptions to remain open and require compensation by active membrane repair mechanisms [10–12]. Several previous studies helped to establish the cellular framework of the plasma membrane repair process. While multiple models of membrane repair have been proposed, most of these models involve exocytotic and/or endocytotic vesicle trafficking to facilitate the resealing of membrane disruptions [13–15]. Given the importance of plasma membrane repair for cell survival, it is likely that most of these putative pathways contribute to the repair process in a cell-type and injury-type dependent fashion.

The current understanding of the plasma membrane repair response comes mainly from studies in striated muscle cells. Much of this interest comes from studies in muscular dystrophies, such as the dysferlinopathies produced by the lack of a membrane repair protein dysferlin [16]. In striated muscle fibers, repair of most sarcolemmal membrane disruptions involves calcium dependent translocation of intracellular vesicles to the injury site where these vesicles then fuse with each other and the plasma membrane, to form a repair patch that restores the integrity of the membrane. This process has several similarities to the release of neurotransmitters from neurons [17]. Given this fact, it is not surprising that proteins involved in vesicle fusion and neurotransmitter release have also been shown to be involved in membrane repair. Inhibition of synaptotagmin I and syntaxin have been used to block membrane repair in axons in the past [18, 19].

Studies from our laboratory group and others demonstrated that mitsugumin 53 (MG53), a muscle-enriched tripartite motif (TRIM) family protein also known as TRIM72, is an essential component of the cell membrane repair machinery in multiple cell types, including striated muscle, liver, and alveolar epithelial cells [4–6, 20–22]. TRIM72/MG53 is an essential component of the membrane repair machinery as TRIM72/MG53 ablation results in defective membrane repair, progressive skeletal myopathy, and vulnerability to ischemia-reperfusion injury (5, 20). Interestingly, when TRIM72/MG53 is expressed in non-muscle cell types it can still function in a similar fashion to increase membrane repair in non-muscle cells [23]. Moreover, exogenously applied recombinant human MG53 (rhMG53) can increase membrane repair and the integrity of muscle and non-muscle cells both *in vitro* [23] and *in vivo* [8, 23]. Specific evidence that rhMG53 is effective in treating injuries comes from studies that efficaciously treated mouse models of muscular dystrophies [23, 24]. Similar to results seen when overexpressing the protein inside various cell types, rhMG53 also increased membrane repair in non-muscle cells *in vivo*. Mice subjected to acute kidney injury showed decreased effects from ischemia reperfusion injury when treated with rhMG53 [8]. Interestingly, rhMG53 has been very successful in treating ischemia/reperfusion injury and has been also used in the treatment of skeletal muscle [25, 26], cardiac muscle [27], and liver tissue [22]. Also, aerosolized rhMG53 has ameliorated the effects of ventilator-induced lung injury [7, 28]. These studies introduce the possibility that rhMG53 could be used to increase the repair capacity of other non-muscle cell types like neurons.

While these previous studies focused on striated muscles identified some of the molecular components of the membrane repair process [29], other studies established that disruption in plasma membrane repair and integrity can occur in many cell types aside from muscle fibers [30–32]. Changes in membrane repair capacity can lead to a number of diseases including heart failure, Alzheimer's disease and neurodegeneration [31, 33–41]. Despite the relevance of membrane repair to these disease states, there has been little investigation of membrane repair specifically in neurons. It is possible that by increasing the membrane repair capacity of neurons, we could potentially minimize the death of these cells and thereby, affect the progression of various neuronal diseases. While rhMG53 has been shown to increase membrane repair capacity [8, 23], the lack of knowledge of the membrane repair process in neurons presents a novel opportunity to explore the potential efficacy of modulating membrane repair in neurons. In this study, we assess the therapeutic potential of rhMG53 in neuronal cells both *in vitro* and *in vivo*. We find that a robust repair response exists in various neuronal cells and that rhMG53 can increase neuronal membrane repair *in vitro*. Additional experiments found that treatment with rhMG53 significantly increased regeneration in an *in vivo* mouse model of sciatic nerve injury. These data indicate that neurons have an endogenous membrane repair response that can be targeted with rhMG53, and further indicates that there are potential therapeutic benefits to elevating membrane repair in neurons that could have protective effects against injuries to the nervous system.

## Results

### TRIM72/MG53 is not expressed in neurons

**Fig 1:**
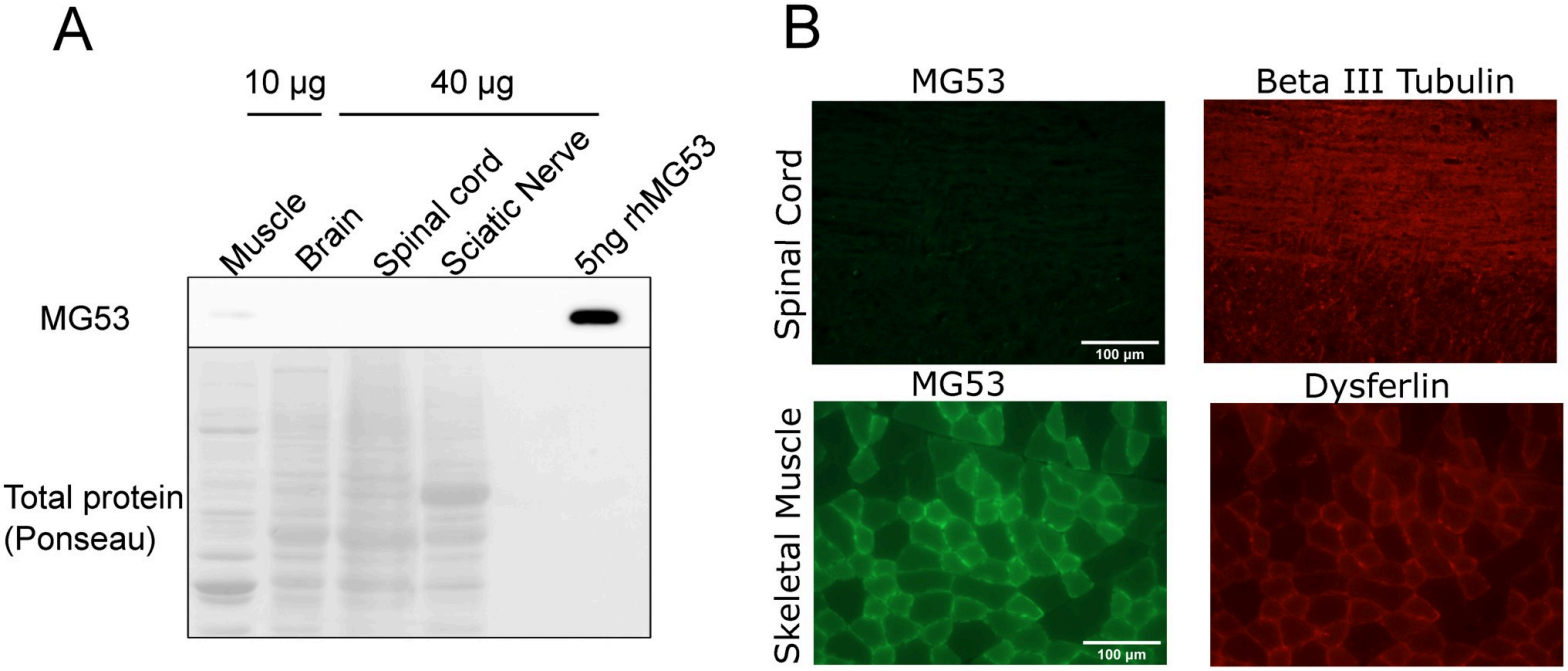
MG53 is not expressed in the nervous system. (A) Lysates from an adult wild type mouse were used for western blotting to detect levels of MG53. One fourth the amount of muscle lysate was used compared to nervous tissue lysate, and no band was detected in the nervous system lysate. Ponceau S stain was used to visualize total protein in lysates (B) Sections from wild type mice were analyzed for the presence of MG53. Cross sections of tibialis anterior muscle was positive for MG53 expression, and dysferlin was used as a counterstain. Longitudinal sections of spinal cord immunostained for MG53 show no MG53 expression, and with beta III tubulin was used as a counterstain.

Initial TRIM72/MG53 studies indicated that it was expressed exclusively in the striated muscle tissues of the skeletal muscle and the heart [20]. Further examination showed that TRIM72/MG53 expression might appear exclusively in skeletal muscle in humans [42]. While TRIM72/MG53 does appear to be highly enriched in striated muscle, recent studies indicate that under certain conditions, TRIM72/MG53 expression can be found in certain cell populations in non-muscle tissues. TRIM72/MG53 expression has been shown in lung type II alveolar epithelial cells where it is important for resisting mechanical injury to the lung [7]. While the liver does not usually express TRIM72/MG53, it appears that ischemia can induce expression in this tissue [22]. Thus, we conducted studies to determine if TRIM72/MG53 was expressed in neurons. Western blot analysis was used to examine mouse tissues from the central nervous system (whole brain and spinal cord lysates) and the peripheral nervous system (sciatic nerve lysate). TRIM72/MG53 expression was not observed in any of these neural tissues when compared to the positive control of skeletal muscle lysate (Fig 1A).

Since previous studies indicate that TRIM72/MG53 may be expressed in subpopulations of cells in a given tissue, we used immunohistochemistry to test for expression of TRIM72/MG53 in the mouse spinal cord (Fig 1B). No TRIM72/MG53 expression was observed in individual cells seen in these sections when compared to the positive control of mouse skeletal muscle. These results from several different areas of the nervous system establish that TRIM72/MG53 is not detected in the peripheral or central nervous system.

### Membrane repair responses are active in cultured neurons

**Fig 2:**
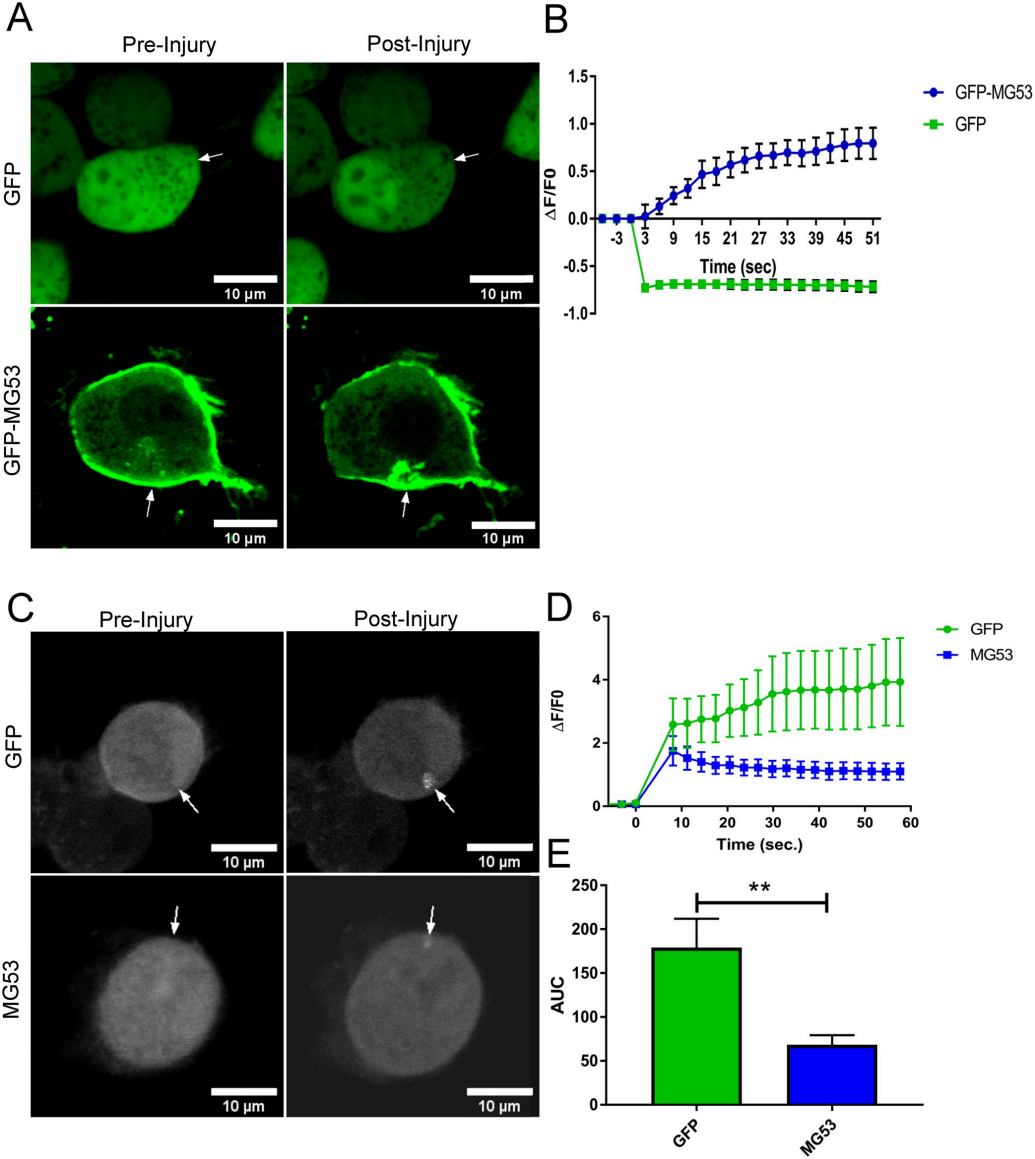
MG53 increases membrane repair capacity in N2a cells. (A) A multiphoton microscope was used to injure transfected Neuro2a cells transfected with GFP or GFP tagged MG53. After injury, the GFP signal can be seen moving to the injury site in the GFP-MG53 cells, whereas no signal was detected in the GFP control cells (B) Graphical representation of change of fluorescence at the injury site over time indicates that GFP-MG53 accumulates at the injury site over the span of 1 minute, while the GFP injured cells become photobleached and fail to recover their signal at the site of injury (n = 8 per group). (C) In the presence of lipophilic dye (FM 4–64) Neuro2a cells transfected with GFP or co-transfected with GFP and MG53 –MBP were injured with a multiphoton microscope. Representative images show dye accumulating at the injury site and the change in fluorescence graph indicated that MG53 transfected cells show reduced dye intake when compared to GFP control. (D, E) Traces were analyzed using the area under the curve (AUC), and compared using a t-test (n = 12 per group, ** = p>.001).

While previous studies examined the cellular response in severed axons that likely make use of membrane repair responses [43, 44], there have been limited studies of the membrane repair response in the cell body of neurons. We examined whether cell membrane disruptions in the neuron body lead to the formation of a typical membrane repair patch as seen in other cell types following membrane repair. To visualize the formation of a membrane repair patch, we transfected Neuro2a (N2a) cells with a GFP tagged TRIM72/MG53 construct (GFP-MG53) that has been shown to translocate to the site of membrane repair patches by multiple laboratory groups [20, 45]. Transfected N2a cells were injured using a multiphoton infrared laser and the localization of GFP-MG53 or GFP alone (as a control) were monitored using confocal microscopy. We observed that following membrane injury the GFP-MG53 moves rapidly to the injury site while GFP remains diffusely localized throughout the cytoplasm (Fig 2A). Quantification of GFP fluorescence at the site of injury from multiple cells (Fig 2B) shows that GFP is bleached at the injury site and that there is no rapid recovery of GFP fluorescence at that site. In contrast, the GFP-MG53 rapidly translocates to the injury site with the fluorescent signal reaching a plateau at approximately 30 seconds after the cell is injured. These results indicate that a membrane repair patch can effectively form at injury sites in the cell body of neuronal origin cells in a manner to that previously observed in muscle cells.

### Cultured neurons display effective membrane repair

While we observed that neural origin cells can form a membrane repair patch, those experiments are not able to resolve if that patch is effective at restoring the integrity of the membrane following injury. To test if the membrane repair patch is functional, we used an FM4-64 dye exclusion assay to determine how effective the membrane repair response is in these cells. The lipophilic dye FM4-64 fluoresces poorly in aqueous solution when outside the cell but provides a strong fluorescent signal when bound to lipids within the cell membrane or the cytosol. When multiphoton confocal laser microscopy is used to injure the membrane, the FM4-64 dye enters the cell and the fluorescent signal increases until the membrane reseals. Less dye influx corresponds to more efficient membrane repair in the cell.

N2a cells were transfected with GFP-MG53 or GFP and then injured by the infrared laser (Fig 2C). Quantification of the extent of FM4-64 dye entry in multiple cells shows that N2a cells can effectively reseal their membrane as the dye influx will rapidly stabilize within 30 seconds of the injury. We also find that transfection of GFP-MG53 accelerates membrane repair in N2a cells as it does in other non-muscle cell types [23]. These results indicate that neural origin cells intrinsically display membrane repair in their cell bodies and that this membrane repair can be accelerated by expression of TRIM72/MG53.

### MG53 increases the membrane repair capacity of cultured neuronal cells

**Fig 3:**
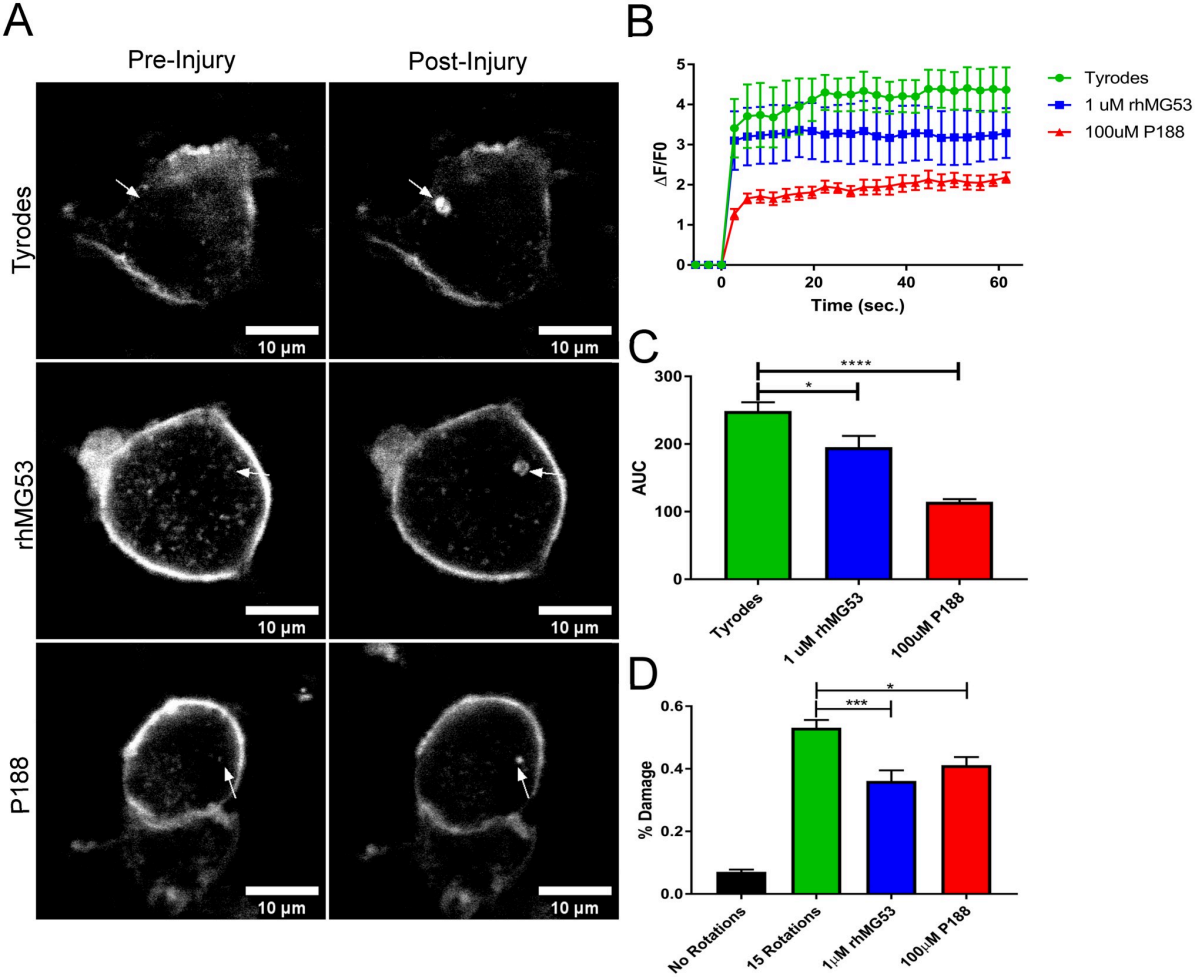
rhMG53 decreases membrane damage *in vitro*. (A-C) Dorsal root ganglion neurons were cultured for 5 days, and then incubated with a lipophilic dye (FM 4–64) and injured in the presence of 1 μM rhMG53, 100μM P188, or control using a multiphoton microscope. (A) Representative images before and after the injury event visualize dye influx at the site of injury. (B, C) Traces marking the change in fluorescence over time graph indicated that rhMG53 and P188 treated cells show reduced dye intake when compared to control. Traces were analyzed using the area under the curve (AUC), and compared using a t-test (*p<0.1, ***p<0.001, n = 8 per group). (D) Rotation damage assay using 100μm glass beads to damage Neuro2a cells. rhMG53 treated cells have reduced LDH leakage, and thus greater resealing and mitigation of injury. (*p<0.1, ***p<0.001 n = 12 per group).

Given that neural cells display an effective membrane repair response and that this membrane repair response can be accelerated by expressing TRIM72/MG53, we tested if rhMG53 can increase the membrane repair capacity in primary isolated neurons, and cultured neural origin cells. We first tested if treatment with rhMG53 before laser injury could improve membrane repair in isolated mouse dorsal root ganglion (DRG) neurons (Fig 3A). We find that low dose rhMG53 (1 μM) can increase membrane repair by significantly decreasing the influx of FM4-64 dye (Fig 3B). We also find that the use of another membrane resealing agent, poloxamer 188 (P188) [46, 47], also increases membrane repair when applied to cultured DRG neurons (Fig 3C), albeit when provided at a high dose (100 μM). These results show that primary neurons have a membrane repair response and that it can be improved through application of known membrane resealing agents.

While laser based injury is widely used to assess membrane repair capacity [20, 48–50] we determined if other methods of neuronal injury could be affected by rhMG53. We used an assay that mechanically injured N2a cells with glass microbeads. In this assay, the amount of intracellular lactate dehydrogenase (LDH) that leaks into the extracellular space gives an indication of the extent of membrane repair in the cell population; less LDH release indicates more effective membrane repair. Application of rhMG53 (1 μM) can improve membrane repair to the same extent as a high dose of P188 (100 μM) following mechanical injury to N2a cells (Fig 3D). These findings indicate that neuronal cells can reseal following mechanical injury and that these resealing agents can improve membrane repair resulting from other sources of injury.

### rhMG53 increases regeneration after sciatic nerve crush injury *in vivo*

**Fig 4:**
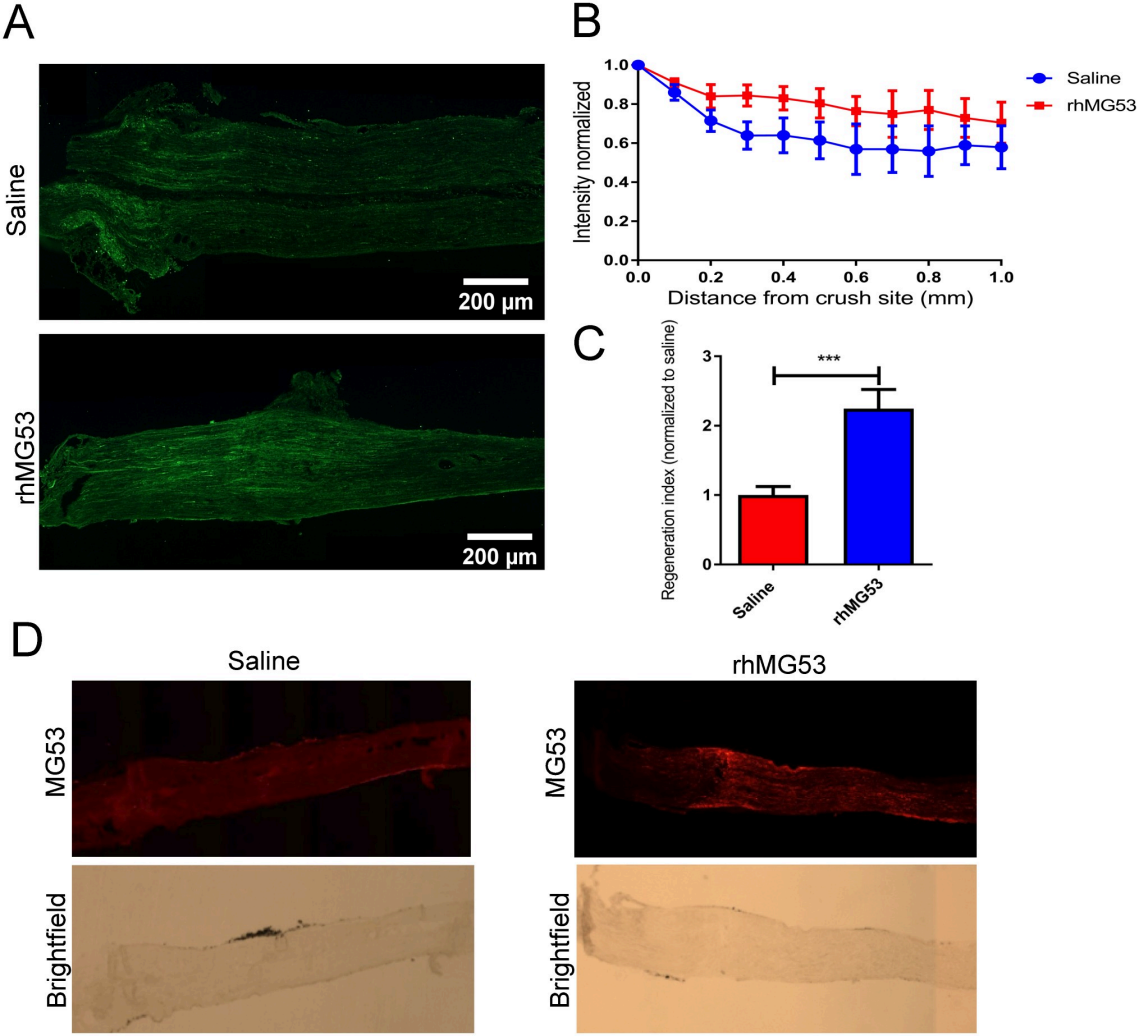
Treatment with rhMG53 significantly increases regeneration past the crush site. A) Longitudinal sections of sciatic nerves from mice that received a crush injury and saline or rhMG53 were immunostained with SCG10. Treatment with rhMG53 increased SCG10 intensity distal to the crush site by 3 dpi. Results are expressed as mean ± SEM. Significant for treatment (***p = 0.0006) and distance (**p = 0.009) by two-way ANOVA. B, C) Treatment with rhMG53 increased the regeneration index. Results are expressed as mean ± SEM, ***p = 0.001, unpaired *t* test (n = 4 per group). D) Longitudinal sections of sciatic nerves from mice that received a crush injury and saline or rhMG53 were immunostained with MG53. rhMG53 treated nerves were observed to stain positive for MG53 distal to the crush site where injection occurred.

Since we observe that isolated neurons display membrane repair and that this repair response can be improved with the application of rhMG53, we wanted to establish if there could be therapeutic effects *in vivo*. To test this, we determined if rhMG53 can be effective at mitigating damage to allow for increased nerve regeneration in a mouse model of sciatic nerve crush injury. C57Bl/6J mice were anesthetized, and the sciatic nerve was surgically exposed to allow for a 10 second mechanical crush injury. Immediately following the crush injury, 1μL of rhMG53 (1mg/mL) or saline vehicle control was injected into the epineurium distal to the crush site. At 3 days post-injury, the sciatic nerve was removed and fixed in paraformaldehyde then immunostained for SCG10, a marker for regenerating axons [51, 52] (Fig 4A). The intensity of SCG10 staining was quantified along the length of the sciatic nerve. rhMG53 treated nerves were observed to have regenerating axons extending further than the saline treated nerves (Fig 4B). A regeneration index was generated by calculating the distance from the crush site at which the average intensity of SCG10 staining was half that at the crush site [53–55]. There was a two-fold increase in regeneration of rhMG53 treated nerves when compared to saline treated (Fig 4C). These findings indicate that rhMG53 treatment significantly increased regeneration in the sciatic nerves compared to saline treated control nerves. This effect can be directly linked to the presence of rhMG53, because at 3 days post-injury, rhMG53 was observed by immunocytochemistry in the rhMG53 treated nerves while no MG53 immunostaining signal appeared in the saline treated nerves (Fig 4D). Thus, the presence of rhMG53 in the nerve correlates with an increased survival of neurons that allows for more robust regeneration at the crush injury.

## Discussion

Our studies show that neurons have an endogenous membrane repair mechanism that is capable of forming a membrane repair patch in the cell body in response to various forms of cell injury. These membrane repair patches are capable of restoring the barrier function of the plasma membrane [17, 56]. We also find that overexpression of TRIM72/MG53 or the application of rhMG53 can increase the membrane repair capacity of neurons *in vitro* and *in vivo*. This capacity for rhMG53 to increase membrane repair in neurons translated to increased regeneration in a mouse model of sciatic nerve injury. Since rhMG53 can compensate for mechanical injury of N2a cells and laser injury of DRG cell bodies by increasing membrane repair, we interpret this finding to mean that application of rhMG53 to the injured nerve could potentially lead to increased cell survival and/or an overall decrease in the damage done to neurons by the crush injury, which leads to increased regeneration or more effective regeneration. This hypothesis is supported by a growing list of evidence showing the benefits of rhMG53 as a facilitator of membrane repair to treat various cellular injuries [7, 8, 25, 27, 57], including ischemia/reperfusion, chemical, and damage resulting from genetic mutations such as in muscular dystrophy mouse models.

Our results suggest that rhMG53 could potentially be used for a variety of neurotrauma events and potentially various neurodegenerative diseases. We show that rhMG53 can be effective in the neuron cell body where it could contribute to the survival of the whole cell and not just to projected axons. This makes rhMG53 a potential treatment for a neurotrauma like traumatic brain injury, spinal cord injury, or disruption of peripheral nerves. Outside the direct increase of membrane repair capacity, there may be additional effects of rhMG53 that could be beneficial in the treatment of neural injury. Injuries to the central nervous system are exacerbated by various inflammatory responses of the immune system [58, 59]. Previous studies suggest that following lipopolysaccharide-induced neural toxicity, the application of rhMG53 reduced microglial activation [60]. While an exact mechanism for this effect is not known, this capacity may allow rhMG53 to both dampen the immune response, as well as increase membrane repair, which would increase its value as a therapeutic for treating injuries to the nervous system. This concept is supported in a rat stroke model, where rats subjected to a brief ischemia/reperfusion event and then treated with rhMG53, show a smaller infarct area [61]. That study and our nerve crush model both showed efficacy when rhMG53 was applied immediately post-injury. This is an important finding because it shows that there is potential for the use of rhMG53 as a therapy since treatment for an injury must be effective after an injury occurs. Glial cells play an important role the regeneration process [62], which could lead to future studies investigating if there are benefits to increasing membrane repair in glial cells or other non-neuron cell types in the nervous system.

We were unable to find TRIM72/MG53 protein expression in any of the neural tissues that we examined. While there is no native TRIM72/MG53 in these tissues, the overexpression of TRIM72/MG53 or delivery of rhMG53 can increase membrane repair. This suggests that TRIM72/MG53 is interacting with conserved membrane repair machinery present in many different cell types to accelerate the membrane repair process when it is necessary to improve membrane repair and allow for cell survival. It is also possible that there are other proteins that function in membrane repair in neurons that have yet to be discovered. Future studies of the neuron membrane repair process will help to determine if there are other proteins that can be linked to this process in neurons.

While our results and previous studies show that rhMG53 can increase membrane repair capacity and reduce the effects of injury, the long-term effects of treatment with this protein are unknown. Recent studies suggest that circulating levels of native TRIM72/MG53 protein can affect insulin sensitivity and metabolic homeostasis [63]. These potential issues would be less of a concern for treating neural injuries where there is an acute injury that does not require prolonged application of the protein. In any case, it may prove to be more beneficial to target the endogenous membrane repair mechanism in neurons through different means. Our results, supported by other studies [64], also indicate that P188 can be effective at increasing neural membrane repair, so it may represent another potential therapeutic approach.

Our study focuses on the impact of increasing the membrane repair capacity in the nervous system. We used rhMG53 to increase the repair capacity in neurons and found that increasing membrane repair in a sciatic nerve crush model extended the length of regenerating neurons. This could address multiple unmet needs in treating diseases and injuries to the nervous system so future studies will address the efficacy of such approaches.

## Materials and methods

### Mice

C57Bl/6J were bred and maintained in standardized conditions at 22 ± 2C under a 12-hr/12-hr light cycle (lights on at 7 a.m. EST). All experimental procedures were approved by The Ohio State University Institutional Animal Care and Use Committee. Animals were maintained in accordance with the recommendations of the NIH Guide for the Care and Use of Laboratory Animals.

### Western blotting

Tissue was taken from adult mice and extracted using Radioimmunoprecipitation Assay buffer (RIPA; Cell Signaling Technology, Danvers, MA, USA). Protein concentrations were determined in accordance with the standard Bradford Assay using bovine serum albumin (BSA) standards. Protein samples (10μg/lane skeletal muscle; 40μg/lane spinal cord, brain, and sciatic nerve; 5n g/lane rhMG53) were separated by SDS-PAGE at room temperature on 10% gels at 150 V and were transferred to nitrocellulose membranes (Bio-Rad, Hercules, CA, USA). Blots were stained with Ponceau S (Boston Bioproducts, Ashland, MA, USA) to visualize total protein. Blots were probed for TRIM72/MG53 with a custom polyclonal antibody (Pacific Immunology, San Diego, CA, USA), and anti-rabbit horseradish peroxidase (HRP)–conjugated secondary antibodies (Cell Signaling Technology). The blots were developed using enhanced chemiluminescence (ECL) substrate (Bio-Rad). An Azure Biosystems imager was used to visualize chemiluminescent blots.

### Membrane damage assays

#### Laser injury

Neuro2a (American Type Culture Collection (ATCC), Manassas, VA, USA) cells were cultured in DMEM (SIGMA, St. Louis, MO, USA) containing 10% FBS (VWR International, Radnor, PA, USA), 1x penicillin-streptomycin-glutamine (Life Technologies, Carlsbad, CA, USA). Membrane damage was induced in Tyrode’s solution with 2.0 mM Ca^2+^, using the Olympus FV1000 multi-photon laser scanning confocal system. For laser injury measurements, injury was induced in the presence of 2.5 μM FM4-64 fluorescent lipophilic dye (Life Technologies). rhMG53 protein was dissolved in saline solution and used at 1μM concentration. A circular area was selected along the edge of the cell membrane and irradiated at 20% laser power for 5 s. Pre- and post-damage images were captured every 3 s, continuing for 57 s. The extent of membrane damage was analyzed using ImageJ software, by measuring the fluorescence intensity encompassing the site of damage. To preclude any potential for bias, all of the experiments were performed in a blinded fashion.

Mouse cervical, thoracic, and lumbar DRG neurons were dissected from CO2 asphyxiated adult mice and dissociated using Dispase ll (10mg/ml; SIGMA) and Collagenase type 1 (500 μg/ml; Worthington Biochemical Corp., Lakewood, NJ, USA) for three separate 45min incubations at 37°C. Next, DRG were triturated in 0.5 ml of HBSS media, and centrifuged at 3000 rpm for 3 min. The neuron-enriched pellet was resuspended in 0.1 ml of primary neuronal growth media (LONZA, Basel, Switzerland). Neurons were plated onto coverslips pre-coated with poly-L-lysine (0.1 mg/ml; SIGMA) and laminin (10 μg/ml; SIGMA). DRG neurons grew for 5 days at 37°C in a 5% CO2 humidified incubator. After 5 days the neurons were treated as described above for laser injury.

#### Rotation damage assay

Using aseptic techniques, 1x10^5^ Neuro2a cells were plated into 2mL micro-centrifuge (VWR International) tubes and incubated at 37° and 5% CO_2_ for 18 hours in 500μL complete medium. After the cells adhered to the bottom of the tube the cells were washed with 200μL PBS. Following the initial wash, 200μL of 2mM Ca2+ Tyrode’s solution and 20μL of ≤106μm glass beads (~4.20 mg) (SIGMA) were added to the culture tubes. All tubes were sealed with parafilm and rotated 360°, 15 times, ~4 seconds per revolution, using a Fisher Scientific Hematology/Chemistry Mixer 346. Following these rotations, 10μL of the supernatant from each sample was transferred to a 96 well plate with technical duplicates. Lactate dehydrogenase (LDH) levels in these samples were determined using a commercial colorimetric assay kit per manufacturer’s instructions (Takara, Japan; MK401). A set of tubes were lysed using 2% Triton X-100 (Sigma) to provide a maximum LDH release level that was used to normalize the data. All experimental conditions were performed in triplicate. For a no damage control glass beads were not added to the sample and the tubes were not inverted.

### Immunostaining procedures

Mice were euthanized by use of CO_2_ asphyxiation followed by cervical dislocation, and spinal cord and tibialis anterior muscles were extracted. Tissue was then fixed with 10% phosphate buffered formalin, followed by a 24 hour incubation with 70% ethanol. Tissue was then embedded in paraffin (Thermo) and 12μm sections were mounted on SuperFrost Plus slides (Fisher Scientific, Hampton, NH, USA). Slides were deparaffinized through changes of xylene and rehydrated through decreasing concentration of ethanol. Antigen retrieval was then performed using Citra Plus Solution (Biogenex, CA, USA). Slides were rinsed with PBS and incubated with a 2.5% BSA (SIGMA) blocking solution for 1 h. Sections were then washed and incubated overnight with TRIM72/MG53 (custom rabbit polyclonal antibody generated Pacific Immunology) (24), dysferlin (Leica Biosystems, IL, USA), beta-III Tubulin (Novus Biologicals, Littleton, CO, USA) and a 488-conjugated goat anti-rabbit IgG antibody, 568-conjugated goat anti-chicken IgG antibody, or 568-conjugated goat anti-mouse IgG antibody (Life Technologies).

Mice were euthanized and perfused by transcardial perfusion with PBS followed by 4% paraformaldehyde in PBS. Nerves were post-fixed in paraformaldehyde for two hours, rinsed in PBS, and then stored in 30% sucrose in PBS at 4°C. Sciatic nerves were embedded in optimal cutting temperature compound (OCT; VWR International) and frozen at -80°C; 12μm sections were cut using a cryostat and thaw-mounted on SuperFrost Plus slides (Fisher Scientific), then stored at -20°C until use. After drying at RT°, slides were rinsed with PBS and incubated with a 2.5% BSA (SIGMA) blocking solution for 1 h. Sections were then washed and incubated overnight with TRIM72/MG53 (Pacific Immunology), SCG10 (Novus Biologicals), and a 488-conjugated goat anti-rabbit IgG antibody, or 568-conjugated goat anti-rabbit IgG antibody (Life Technologies).

### Surgical procedures

Four month old male C57Bl/6 mice were anesthetized (80 mg/kg ketamine; 10 mg/kg xylazine) then, following shaving and aseptic preparation of the right hind leg, a Dumont #5 forceps was used to crush (10 s duration) the sciatic nerve. The site of nerve injury was marked by charcoal. 1μL of rhMG53 (1mg/mL) or saline vehicle control was injected into the epineurium, distal to the crush site. The 1mg/ml dose was selected to produce these therapeutic levels in the target tissue [23] by accounting for dilution in the endoneurial fluid, leakage from the crush site, and quick diffusion at the crush site. The muscle/fascia layer was pulled together and the skin was sealed with a single wound clip. Three days after crush injury, mice were perfused, and the sciatic nerves were harvested and prepared for histology as described above.

## Supporting information

S1 Raw images (PDF)
