# Supplementary material for: Enhancing membrane repair increases regeneration in a sciatic injury model
Source: PLoS One. 2020 Apr 9;15(4):e0231194. doi: 10.1371/journal.pone.0231194 (PMC7145019; doi:10.1371/journal.pone.0231194)

Original blot probed for MG53 imaged on an Azure Biosystems imager with ECL substrate

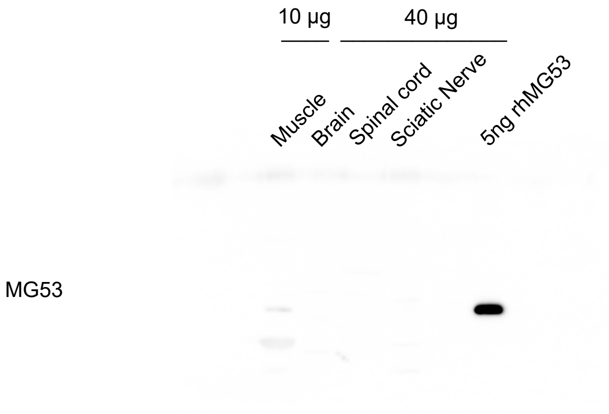

Original blot probed for MG53 imaged on an Azure Biosystems imager with ECL substrate. Marker overlayed in ImageJ

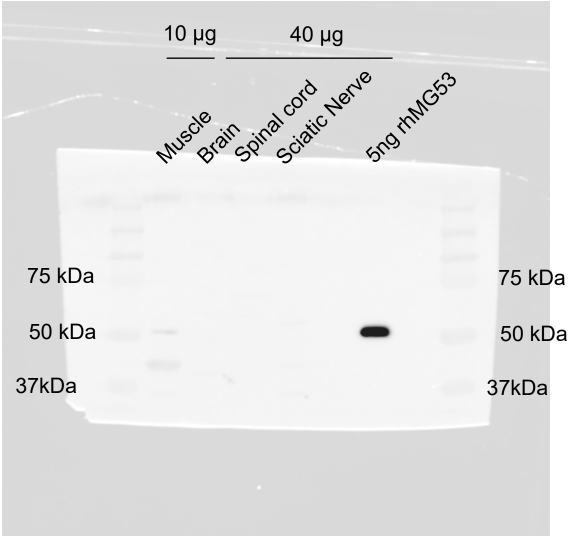

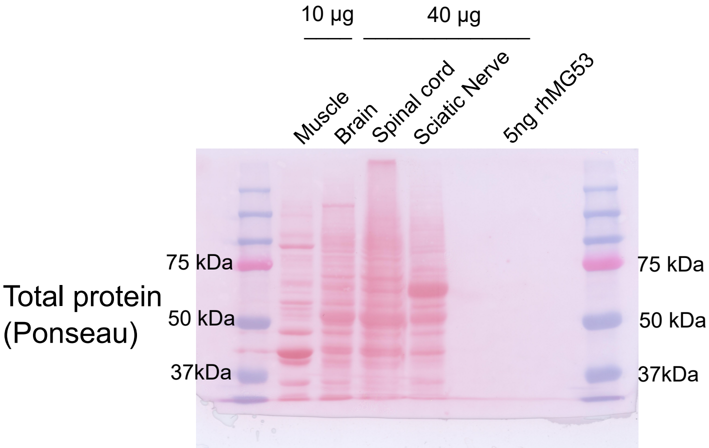

Supplement: S1 Raw images — (PDF) [file pone.0231194.s001.pdf]
